# Characterization of Three *Fusarium graminearum* Effectors and Their Roles During Fusarium Head Blight

**DOI:** 10.3389/fpls.2020.579553

**Published:** 2020-11-30

**Authors:** Guixia Hao, Susan McCormick, Thomas Usgaard, Helene Tiley, Martha M. Vaughan

**Affiliations:** USDA, Agricultural Research Service, Mycotoxin Prevention and Applied Microbiology Research Unit, National Center for Agricultural Utilization Research, Peoria, IL, United States

**Keywords:** *Fusarium graminearum*, effectors, gene expression, *Fusarium* head blight, reactive oxygen species, immunity

## Abstract

*Fusarium graminearum* causes Fusarium head blight (FHB) on wheat, barley, and other grains. During infection, *F. graminearum* produces deoxynivalenol (DON), which contaminates grain and functions as a virulence factor to promote FHB spread throughout the wheat head. *F. graminearum* secretes hundreds of putative effectors, which can interfere with plant immunity to promote disease development. However, the function of most of these putative effectors remains unknown. In this study, we investigated the expression profiles of 23 *F. graminearum* effector-coding genes during the early stage of wheat head infection. Gene expression analyses revealed that three effectors, *FGSG_01831, FGSG_03599*, and *FGSG_12160*, respectively, were highly induced in both a FHB susceptible and a moderately resistant variety. We generated deletion mutants for these effector genes and performed FHB virulence assays on wheat head using point and dip inoculations to evaluate FHB spread and initial infection. No statistically significant difference in FHB spread was observed in the deletion mutants. However, deletion mutants Δ01831 displayed a significant reduction in initial infection, and thus resulted in less DON contamination. To investigate the potential mechanisms involved, these three effectors were transiently expressed in *Nicotiana benthamiana* leaves. *N. benthamiana* leaves expressing these individual effectors had significantly reduced production of reactive oxygen species induced by chitin, but not by flg22. Furthermore, FGSG_01831 and FGSG_03599 markedly suppressed Bax-induced cell death when co-expressed with Bax in *N. benthamiana* leaves. Our study provides new insights into the functions of these effectors and suggests they play collective or redundant roles that likely ensure the successful plant infection.

## Introduction

*Fusarium graminearum*, the causal agent of *Fusarium* head blight (FHB) on wheat and barley, poses a great threat to food safety and food security. In addition to reducing crop yield, this disease results in grain contamination with trichothecenes, predominantly deoxynivalenol (DON). Consumption of contaminated products can have profound effects on animal and human health. *F. graminearum* infects wheat and barley floral structures and results in FHB symptoms including necrosis and bleaching. Typically, visual FHB symptoms appear in inoculated florets 3 days post-infection (dpi). Microscopic observation revealed that *F. graminearum* spores germinated in floral tissue within 12 h post-infection (hpi) and colonized the ovary and floral bracket by 36 hpi. The intercellular hyphae colonized the palea at 2 dpi. Disease symptoms were primarily restricted to the inoculated florets within the first 7 dpi (Brown et al., 2010). This period is considered the initial infection. At 7 dpi, *F. graminearum* starts to reach the rachis and spread to the adjacent florets (Brown et al., 2017). Based on *Fusarium* and host interactions, plant resistance has been classified into two types. Resistance to initial infection is considered type I resistance. Very limited information is available regarding how *F. graminearum* initiates infection and the plant defense mechanisms involved. One recent study showed that type I resistance in barley may be associated with flower surface structures including trichomes and silica/cork cells (Imboden et al., 2018). Resistance to disease spread is called type II resistance. DON functions as a virulence factor that facilitates FHB spread in wheat heads (Proctor et al., 1995; Bai et al., 2002; Jansen et al., 2005). *F. graminearum* mutants blocked in the production of DON are restricted to inoculated florets and cannot pass the rachis barrier to spread to the rest of the wheat head (Jansen et al., 2005). Currently, wheat breeding efforts have been primarily focused on the improvement of type II resistance. Multiple type II resistance quantitative trait loci (QTLs) have been characterized, such as *Fhb1, Fhb2*, and *Fhb7* (Review in Kazan and Gardiner, 2018; Wang et al., 2020). Compared to wheat, barley has a relatively high level of type II resistance. *F. graminearum* typically spreads along the surface of the rachis in barley because it lacks the ability to penetrate through the rachis node regardless of DON production (Maier et al., 2006). Nevertheless, it remains unclear how *F. graminearum* initiates infection and overcomes host immunity to cause disease. Therefore, to enhance FHB resistance in wheat and barley, it is critical to understand the molecular mechanisms involved during initial infection and improve wheat and barley type I resistance.

Effectors play critical roles during pathogen and plant interactions. Broadly, pathogen secreted proteins, secondary metabolites, and toxins are considered effectors. Effectors are typically expressed only after the pathogen encounters the plant tissue. Studies have demonstrated that effectors in fungi with different lifestyles interact differently with plants. During infection, biotrophic pathogens secrete effectors to suppress plant immunity in susceptible hosts or interact with cognate resistant genes to induce programmed cell death (or hypersensitive response) to restrict pathogen spread in resistant hosts. For example, Pep1 (Protein essential during penetration 1) from the biotrophic pathogen *Ustilago maydis* suppresses plant immunity by inhibiting the activity of a secreted maize peroxidase, POX12, which is important for reactive oxygen species (ROS) production (Hemetsberger et al., 2012). In contrast, necrotrophic effectors (also known as host-selective toxins) lead to host susceptibility by inducing cell death to promote disease described as an alternative inverse gene-for-gene model (Friesen et al., 2007). ToxA, an effector produced by the necrotrophic wheat pathogens *Stagonospora nodorum* and *Pyrenophora tritici-repentis*, induces severe necrosis in wheat harboring dominant sensitivity genes (Oliver et al., 2012).

A primary role of effectors is to suppress pathogen-associated molecular patterns (PAMPs) triggered immunity (PTI). ROS production is one of the earliest events responding to pathogen infection during PTI. Many bacterial and fungal effectors have been shown to suppress ROS production induced by PAMPs, such as bacterial flagellin epitope flg22 and/or fungal chitin (Guo et al., 2009; Wang et al., 2011). In Arabidopsis, flg22 is recognized by FLS2 (FLAGELLIN-SENSING 2), which forms an active receptor complex with a co-receptor BAK1 (BRI1-ASSOCIATED KINASE 1) to activate defense responses (Chinchilla et al., 2007). On the other hand, chitin oligosaccharides are perceived by the *Arabidopsis* lysin motif (LysM) RKs LYK5 (LYSIN MOTIF-CONTAINING RECEPTOR-LIKE KINASE 3) and CERK1 (CHITIN ELICITOR RECEPTOR KINASE 1) (Liu et al., 2012; Cao et al., 2014). Studies have shown that transient expression of a mouse protein Bax in tobacco leads to ROS accumulation and disrupts plant plasma membranes, which in turn results in programmed cell death (PCD) (Lacomme and Santa Cruz, 1999). The Bax-induced PCD assay has been widely used to aid in characterizing the function of multiple effectors (Guo et al., 2009; Wang et al., 2011).

*F. graminearum* has a hemibiotrophic lifestyle with two distinct colonization phases: a short biotrophic phase and a ramifying necrotrophic phase (Brown et al., 2017). Bioinformatics analysis predicted that *F. graminearum* secretes about six hundred effectors (Brown et al., 2012). These effectors can modulate plant immunity, suppress defense responses during the biotrophic phase, and induce cell death to promote infection and disease spread during the necrotrophic phase. Many of these effectors are small cysteine-rich proteins (SCPP) that contain N-terminus signal peptides and lack transmembrane domains. Approximately thirty SCPPs have been shown to be expressed during the *F. graminearum*-wheat interactions (Lu and Edwards, 2016). One of them, FGSG_00569, shares homology with the plant pathogenesis-related 1 (PR1) proteins. However, deletion of FGSG_00569 did not affect virulence of *F. graminearum* on wheat heads (Lu and Edwards, 2018). In contrast, the mutants of another secreted PR1-like protein (FGSG_03112) reduced FHB spread by 30% (Lu and Edwards, 2018). Besides, the single or triple mutants of Kp4-like killer toxin proteins, FGSG_00060, 00061, and 00062, did not affect FHB, but reduced seedling rot in some wheat varieties (Lu and Faris, 2019). In addition to SCPP, a few secreted enzymes, such as lipase-like protein FLG1, Tom1, and ARB93B, have been shown to be involved in FHB spread (Voigt et al., 2005; Blümke et al., 2014; Carere et al., 2017; Hao et al., 2019). Our study demonstrated that ARB93B affected FHB development by interfering with ROS associated plant immunity (Hao et al., 2019).

Transcriptomic studies revealed that the expression of many effector-coding genes was upregulated during *F. graminearum* interactions with its host plant (Lysøe et al., 2011; Brown et al., 2017). Furthermore, it was shown that the effector genes were upregulated in asymptomatic and symptomatic tissues, suggesting that these effectors are important for initiating *F. graminearum* infection of the host (Brown et al., 2017). A recent study using laser capture microdissection demonstrated that the expression of 634 *F. graminearum* genes encoding secreted proteins was regulated in infection cushion and running hyphae, of which 328 genes were upregulated during infection (Mentges et al., 2020). Nevertheless, virulence factors essential for FHB initial infection have not been identified yet. Identification of effectors that are essential for FHB pathogenesis will serve as novel targets to mitigate FHB and mycotoxin contamination.

To characterize the effectors critical for *F. graminearum* initial infection, we selected 23 effector-coding genes and determined their expression profiles during the early stages of wheat infection. We generated deletion mutants for three genes, *FGSG_01831, FGSG_03599*, and *FGSG_12160*, which were highly induced during these stages, and evaluated their roles during FHB spread using point inoculation and initial infection using dip inoculations. Furthermore, we transiently expressed these three effectors in *Nicotiana benthamiana* and investigated their potential function during plant and pathogen interactions.

## Materials and Methods

### Culture of Fungal and Bacterial Strains

The wild-type *F. graminearum* strain PH-1 was maintained on V8 juice agar (V8: 113 mL V8 juice, 1.75 g CaCO3, 11.7 g agar, 467 mL H_2_O) with a 12:12 h light/dark cycle at 28°C with ultraviolet light. Mutants were grown on V8 juice agar amended with 150 mg/L hygromycin. *E. coli* and *Agrobacterium* strains were grown in LB with addition of different antibiotics (Kanamycin: 100 mg/L; Zeocin: 50 mg/L; spectinomycin: 100 mg/L).

### Plant Growth Conditions

The wheat cultivars, a susceptible Norm and a moderately resistant Alsen, were used in the experiments. Seeds were surface sterilized with 70% ethanol for 5 min. The ethanol was removed, and 20% bleach was added to treat the seeds for 10 min with occasional inversions. The sterilized seeds were washed three times with sterile water and spread out on damp filter membranes at the bottom of petri dishes for 2 days. The germinated seeds were planted in 7-inch pots. SunShine Mix (Sun Gro Horticulture, Agawam, MA) was used with addition of 100 g Osmocote and 15 g Micromax in 5 L soil. Each pot contained five seeds. The plants were grown in a controlled growth chamber with 16 h light at 23°C and 8 h dark at 20°C with 50% relative humidity. Wheat plants were watered every day and fertilized every 2 weeks with 500 mL solution containing 325 mg/L of Peter's 20:20:20 (Grace-Sierra Horticultural Products, Milpitas, CA) until inoculation.

Seeds of *N. benthamiana* were germinated in a controlled growth chamber with cycles of 14:10 h light/dark at 25°C. Ten-day old seedlings were then transferred into 4-inch plastic pots. One plant was maintained in each pot. Three- to four-week-old *N. benthamiana* plants were used for transient expression.

### Selection of Effector Candidates

Based on previous studies (Brown et al., 2012; Lu and Edwards, 2016), a total of 23 putative effector candidates were selected from the *F. graminearum* genome (Table 1). These effectors contain signal peptides and protein sequences <300 amino acids without predicted transmembrane domains. Many selected effectors are hypothetical proteins and cysteine-rich (Table 1). The N-terminal signal peptides were predicted using SignalP 4.0. The potential localization *in planta* for each effector was predicted by PSORT (https://www.psort.org).

**Table 1:** Characteristics of 23 *F. graminearum* effectors selected for gene expression study.

**Protein**	**Size(aa)**	**Signal peptide(aa)**	**#cys**	**Predicted function**	**Plant target (Certainty)**	**Induction in planta**
FGSG_00060	134	16	10	Kp4 toxin	Cytoplasm (0.7)	N
FGSG_00569	213	16	5	PR1 like-protein	Peroxisome (0.7)	Y
FGSG_01831	98	17	8	Fungal hydrophobin 2	Cytoplasm (0.7)	Y
FGSG_02063	176	21	7	Hypothetical protein	Mitochondria (0.5)	N
FGSG_03581	198	20	4	Hypothetical protein	Cytoplasm (0.5)	N
FGSG_03599	95	18	10	Hypothetical protein-CFEM	Plasma membrane (0.7)	Y
FGSG_04074	190	19	6	Hypothetical protein	Chloroplast/cytoplasm (0.5)	Y
FGSG_04239	206	20	14	Hypothetical protein	Chloroplast/cytoplasm	Y
FGSG_04661	163	17	4	Hypothetical protein	Cytoplasm (0.5)	Y
FGSG_04805	126	23	6	Hypothetical protein	Cytoplasm (0.5)	N
FGSG_05341	189	16	5	Hypothetical protein	Cytoplasm (0.7)	Y
FGSG_05714	201	20	6	Hypothetical protein	Cytoplasm (0.5)	Y
FGSG_06712	146	17	16	Hypothetical protein	Cytoplasm (0.7)	Y
FGSG_06993	200	16	4	Hypothetical protein-HXXP	Peroxisome/cytoplasm (0.5)	Y
FGSG_08210	155	22	10	Hypothetical protein	Cytoplasm (0.6)	N
FGSG_09570	170	19	8	Hypothetical protein	Cytoplasm (0.5)	N
FGSG_10206	162	16	8	Hypothetical protein	Cytoplasm (0.7)	Y
FGSG_11205	140	18	4	Cerato-platanin	Cytoplasm (0.4)	Y
FGSG_11318	153	18	4	Hypothetical protein	Unknown	Y
FGSG_12160	247	16	6	Hypothetical protein	Cytoplasm (0.7)	Y
FGSG_12622	296	18	7	Cyclic nucleosidase	Chloroplast (0.9)	Y
FGSG_13782	97	21	6	Hypothetical protein	Cytoplasm/Chloroplast	Y
FGSG_13952	105	20	10	Hypothetical protein	Cytoplasm (0.7)	N

### Preparation of *F. graminearum* Spores for Inoculation

*F. graminearum* PH-1 and mutant spores were obtained from 4-day old mung bean liquid cultures (Hao et al., 2019). The macroconidia were passed through a 40 μM cell strainer (Biologix, Jinan, Shandong, China) and centrifuged for 10 min at 3,000 g. For point inoculation, the pellet was suspended in water and adjusted to a concentration of 10^5^ conidia/mL. At anthesis, wheat heads were inoculated with 10 μL conidia solution. For dip inoculations, the pellet was suspended in 0.02% Tween 20 (Thermo Fisher Scientific, Waltham, MA, United States) and adjusted to different concentrations for inoculations. To maintain high humidity, the heads were covered in a plastic bag for 3 days after inoculation.

### Inoculation of Wheat Heads for Gene Expression Study

In the first experiment, wheat heads from a susceptible variety, Norm, with 50% of spikelets at anthesis were dipped into a 10^5^ conidia/mL PH-1 suspension in 0.02% Tween 20. Heads were collected at 3, 6, 12, 24, 36 h, and 7 days post inoculation (dpi). In the second experiment, heads from Norm and Alsen, were collected at 1, 2, 3, 4, 5, 6, and 7 dpi. For each time point and each variety, six heads were collected, and two heads were combined as one sample. Three biological replicates were performed for each time point and each variety. All samples were lyophilized, pulverized, and ground for RNA isolation and cDNA synthesis as described (Hao et al., 2019). Briefly, total RNA was isolated, and DNA was digested on a column. First-strand cDNA was obtained from 2 μg of total RNA. Expression analyses of selected effectors were performed using reverse transcription-quantitative polymerase chain reaction (RT-qPCR). Two microliters of the 5-fold diluted cDNA were used in a 20-μL PCR in a CFX96 Real-time PCR Detection system (Bio-Rad, Hercules, CA). Gene-specific primers for each effector gene were designed and listed (Supplementary Table 1). The relative expression level of each gene was normalized by the constitutively expressed β-tubulin gene and was calculated based on the 2^−ΔΔCt^ values (Livak and Schmittgen, 2001). The expression value of cDNA from 7-day-old PH-1 culture from a V8 juice agar plate was set up as one for comparison.

### Generation of Deletion Mutants

The deletion constructs for FGSG_01831, FGSG_03599, and FGSG_12160 were generated using the OSCAR (One Step Construction of Agrobacterium-Recombination-ready-plasmids) method combined with protoplast transformation (Paz et al., 2011; Hao et al., 2019). Briefly, upstream and downstream fragments for each gene were amplified by PCR with primers (Supplementary Table 2). The purified PCR products were introduced into the pOSCAR vector. A BP reaction was performed with pOSCAR plasmids and transformed into *E. coli* competent cells. The correct construct containing the hygromycin (hygB) fragment was confirmed and used for plasmid isolation. Due to the low efficiency of *F. graminearum* PH-1 transformed by *Agrobacterium* strain AGL1, we amplified the PCR products, including the upstream and downstream regions flanking HygB. The purified PCR products were used to transform PH-1 protoplasts. The mutants were selected on V8 juice agar plates containing 150 μg/mL hygromycin B (Sigma, St. Louis, MO). Individual mutants were used for DNA isolation. The absence of target gene was confirmed by OFR-5′ and ORF-3′. Replacement of the target gene with the hygB fragment in the correct location was further confirmed by PCR using two primers, gene-up and Hyg-R210, and Hyg-F850 and target gene-down (Supplementary Table 2).

### FHB Pathogenesis Assay

To examine whether Δ01831, Δ03599, and Δ12160 mutants affected FHB spread, FHB progress was assessed on the susceptible wheat cultivar Norm by single floret inoculation (Hao et al., 2019). Two independent mutant strains were selected for FHB pathogenesis assays (Δ01831-32 and 59, Δ03599- 22 and 23, and Δ12160-10 and 11). At least 16 wheat heads were inoculated for each strain evaluated including wild-type PH-1 and the 6 mutants. Disease was scored by visualized FHB symptoms at 7, 14, and 21 days after inoculation.

Further, these mutants were assessed for FHB initial infection on the wheat variety, Alsen, using dip inoculation. Wheat heads at mid-anthesis were dipped into a 5 × 10^4^ conidia/mL PH-1 suspension in 0.02% Tween 20 as describe above. Disease was scored at 4 and 7 dpi. In the first experiment, mutants Δ01831-32, Δ03599-23, and Δ12160-10 were used for inoculations. In the second experiment, three mutants, Δ01831-32, 40 and 53, were used for inoculations. At least 20 heads were inoculated for each strain. Three heads as a group were pulverized and ground for biomass and DON analysis (Hao et al., 2020). Fungal biomass analysis was performed as described (Hao et al., 2020). Briefly, DNA was isolated from infected tissue (100 mg). The quantity of fungal DNA was determined using *TRI6* gene specific primers (Supplementary Table 1). The quantity of wheat DNA was estimated using GAPDH specific primers (Supplementary Table 1). Relative biomass of *F. graminearum* in the infected tissue was quantified by qPCR. The Ct value for the *F. graminearum* gene *TRI6* was calculated relative to the corresponding Ct values for the wheat gene GAPDH using the 2^−ΔΔ*Ct*^ method (Livak and Schmittgen, 2001). Ten biological replicates for PH-1 and six biological replicates for mutant Δ01831-32 were used in qPCR analysis. Two technical replicates for each sample were performed. Three wheat heads as a group were used for toxin analysis using GC/MS. Means from biological replicates were compared using one-way analysis of variance (ANOVA) and Tukey's honestly significant difference (HSD) *post hoc* test.

### Transient Expression of Selected *F. graminearum* Effectors

To determine the potential function of three highly induced effector coding genes during wheat infection, primers were designed to amplify the coding sequence of the protein with and without the signal peptide (Supplementary Table 2). PCR products for FGSG_01831, 03599, and 12160 were amplified from PH-1 cDNA and cloned into the pDONR™221 vector (Invitrogen, Carlsbad, CA). The obtained positive plasmid for each effector gene was verified by sequencing and then was used for Gateway LR reaction with the vector pGWB411 (Nakagawa et al., 2007). The recombinant plasmids were introduced into the *A. tumefaciens* strain GV2260 by heat shock for a transient expression assay in *N. benthamiana*.

### ROS Production Detection

To examine whether these effectors affect ROS production, ROS assays were performed using a luminol-based chemiluminescence assay as described (Hao et al., 2019). The *A. tumefaciens* GV2260 strain carrying empty vector or individual effectors was adjusted to an OD_600_ of about 0.6 and was then infiltrated into *N. benthamiana* leaves. Leaf discs from infiltrated zones were taken at 2 days post-infiltration (dpi) and floated on water overnight. Prior to ROS measurements, the water was replaced with 200 μL of assay solution [17 mM luminol (Sigma, St. Louis, MO), 1 μM horseradish peroxidase (Sigma, St. Louis, MO), and 100 nM flg22 (AnaSpec, Fremont, CA) or 200 μg/mL crab chitin (Sigma, St. Louis, MO)]. Leaves infiltrated with an empty vector served as positive controls. Leaves without flg22 or chitin treatment served as negative controls. Luminescence was measured for 60 min using the Synergy HT and Gen5 software (BioTek Instruments Inc. Winooski, VT).

### Cell Death Suppression Assay

For co-infiltration assays, each *A. tumefaciens* GV2260 strain carrying Bax, empty vector, or individual effectors was adjusted to an OD_600_ of about 0.8 to 1.0. Bax was co-infiltrated with equal volumes of each effector or empty vector into *N. benthamiana* leaves. The infiltrated zones were monitored for 7 days post-inoculation. Pictures were taken at 5–7 days after inoculation.

## Results

### Selection of Candidate Effectors

Based on previous bioinformatics and proteomics studies (Brown et al., 2012; Lu and Edwards, 2016) and our analysis, we selected 23 putative effectors for investigation. These effectors contain N-terminal signal peptides and lack transmembrane domains. The protein size, signal peptide, potential functional domain, and predicted localization *in planta* of these effectors are listed in Table 1. Although many selected effector candidates are hypothetical proteins, some of them contain conserved functional domains associated with pathogenesis that have been reported in other fungal pathogens. For example, FGSG_03599 contains a CFEM (Common in Fungal Extracellular Membranes) domain. CFEMs have been reported to act as cell-surface receptors, signal transducers, or adhesion molecules during plant-pathogen interactions (Kulkarni et al., 2003).

### Expression of Effector-Coding Genes During Wheat Head Infection

**Figure 1:**
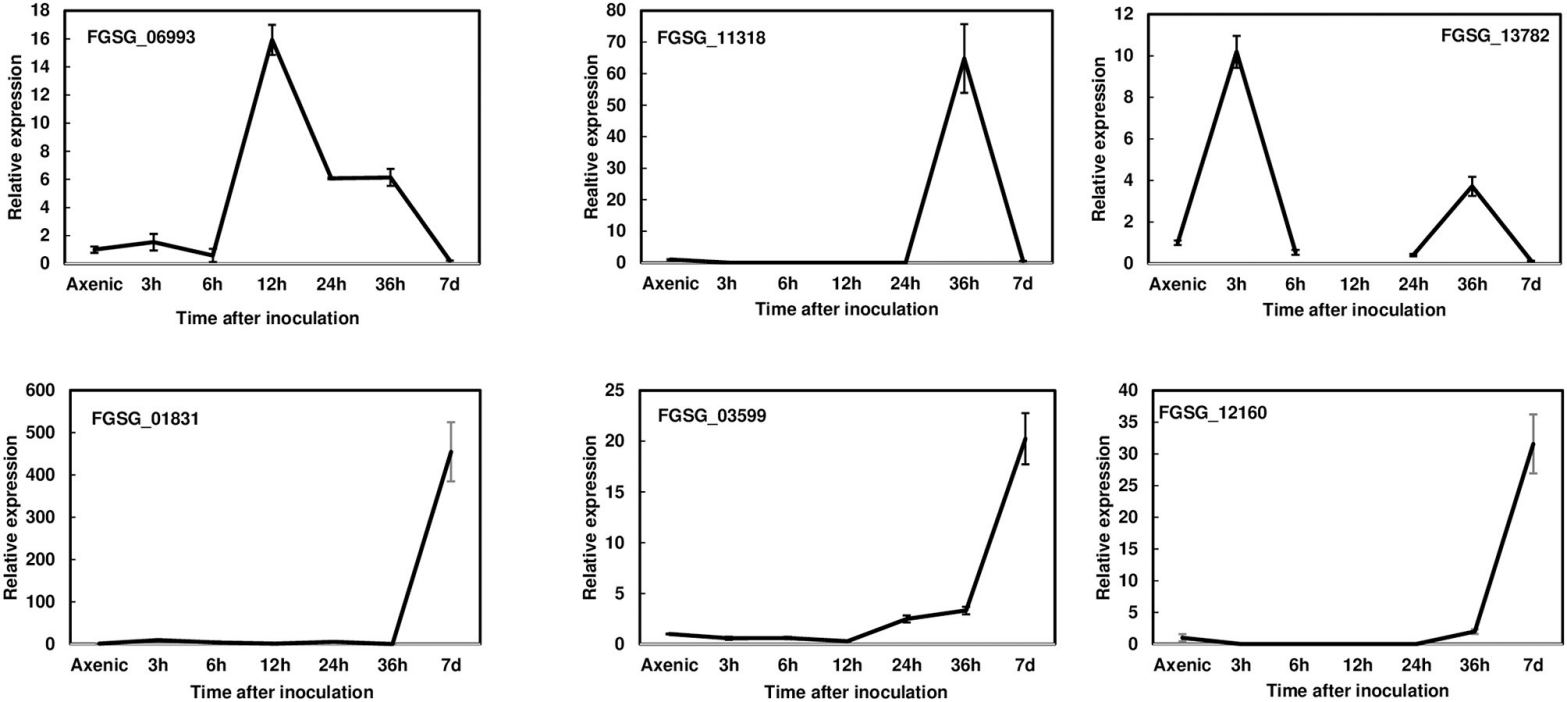
Gene expression profiles of selected effectors upregulated during wheat head infection. Wheat heads were collected at 3, 6, 12, 24, 36 hpi, and 7dpi after whole-head dip inoculation. Three biological replicates, each replicate containing two heads, were collected at each time point and used for RNA isolation and cDNA synthesis. Fungal β-tubulin was used as an internal control for transcript normalization. Fold changes of gene expression were relative to axenic culture, which was grown on V8 plates for 7 days. Three technical replicates were used for each sample.

To reduce the number of candidate genes, we decided to prioritize the characterization of effectors induced during pathogenesis. Thus, we first examined the expression profiles of 23 putative effectors during wheat head infection. We dip inoculated Norm wheat heads with the *F. graminearum* strain PH-1 and collected samples at 3, 6, 12, 24, 36 hpi, and 7 dpi for gene expression analysis by RT- qPCR. These genes were classified into three categories based on their expression profiles: (1) the immediate-early stage of induction (peaked before 36 hpi); (2) the early stage of induction (induced after 36 hpi); and (3) expressed but not induced in the infected tissue in comparison with axenic culture. We found that the expression of six effector genes was induced at the immediate-early stage (Figure 1, Table 1). The expression of *FGSG_06993* was detected at 3 hpi, increased 14-fold at 12 hpi, and decreased after 24 hpi. Contrastingly, the expression of *FGSG_11318* displayed the highest level at 36 hpi (60-fold) but had decreased dramatically at 7 dpi. Interestingly, the expression of *FGSG_13782* showed two small peaks at 3 hpi (10-fold) and 36 hpi (5-fold) (Figure 1). The expression of *FGSG_01831, FGSG_03599*, and *FGSG_12160* was first detected at 36 hpi and displayed high expression at 7 dpi (Figure 1). In addition, the expression of *FGSG_04074, FGSG_04661, FGSG_05714*, and *FGSG_11205* were induced <10-fold (Table 1). On the other hand, several effector genes, such as *FGSG_08210* and *FGSG_13952*, were highly expressed in axenic culture but were down regulated during wheat head infection (Table 1, Supplementary Figure 1). The remaining candidates did not show significant differences between axenic culture and during plant infection (Table 1).

**Figure 2:**
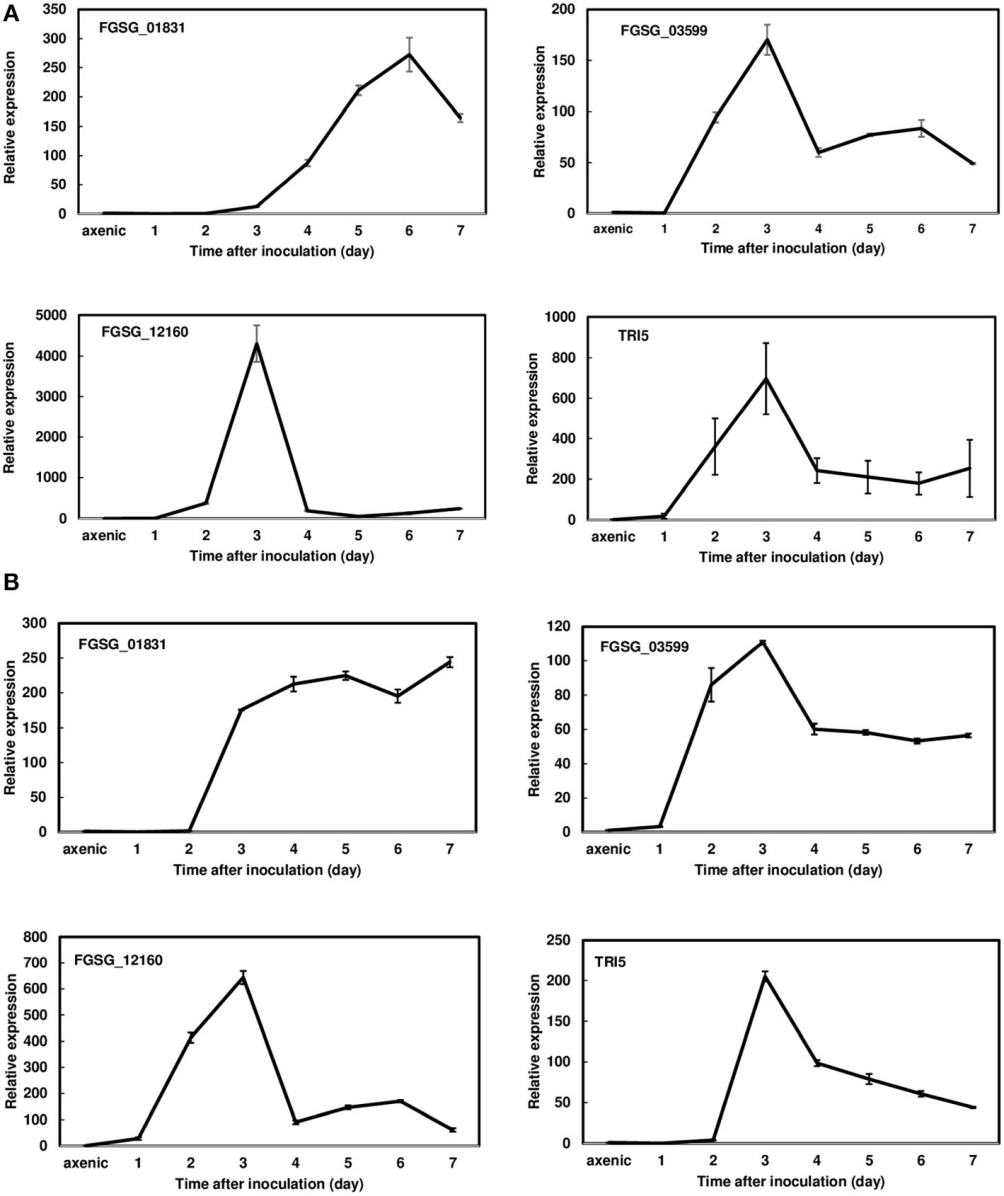
Gene expression profiles of *FGSG_01831, 03599, 12160*, and *TRI5* from 1 to 7 days post inoculations (dpi) during wheat head infection. **(A)** Norm; **(B)** Alsen. Wheat heads were collected at 1, 2, 3, 4, 5, 6, and 7 dpi. Three biological replicates, each replicate containing two heads, were collected at each time point and used for RNA isolation and cDNA synthesis. Gene expression was determined by RT-qPCR. Fungal β-tubulin was used as an internal control for transcript normalization. Fold changes of gene expression were relative to axenic culture, which was grown on V8 plates for 7 days. Two technical replicates were used for each sample.

Since *FGSG_01831, FGSG_03599*, and *FGSG_12160* displayed a similar induction pattern between 36 hpi and 7 dpi, we further examined the expression profiles of these three effectors in a susceptible variety, Norm, and a moderately resistant variety, Alsen, every 24 h for the first 7 dpi. During this period, *F. graminearum* was primarily colonized in the inoculated florets showing typical FHB symptoms. Expression analysis revealed that these three effectors were highly induced in both varieties during the initial infection period evaluated. In Norm, the expression of *FGSG_01831* was induced at 2 dpi, steadily increased, peaked at 6 dpi (272-fold), and then decreased (Figure 2A). Whereas in Alsen, *FGSG_01831* expression was induced at 2 dpi and remained elevated throughout the time course (Figure 2B). The expression of *FGSG_03599* was similarly induced in Norm and Alsen, initiated at 1 dpi, peaked at 3 dpi, decreased, and then stayed at a similar level from 4 to 7 dpi. For comparison, the expression of *TRI5*, the first gene involved in DON biosynthesis, displayed a similar induction profile as *FGSG_03599* (Figure 2). *FGSG_12160* was induced at 2 dpi, sharply increased to 4,300-fold at 3 dpi, and decreased dramatically between 3 dpi and 4 dpi in Norm (Figure 2A). Although the induction of *FGSG_12160* displayed a similar pattern in Norm and Alsen, a higher expression was observed in Norm compared to Alsen (Figure 2). In addition, we examined the expression of the effectors that were suppressed during the first-time course analysis and found that none were induced during 1 to 7 dpi.

### Generation of Deletion Mutants

Based on the gene expression analyses we selected *FGSG_01831, FGSG_03599*, and *FGSG_12160* for further functional characterization. We generated deletion mutants for these genes to determine their potential roles during *F. graminearum* infection. A modified OSCAR protocol was used to generate mutants. After protoplast transformation and PCR screening, multiple deletion mutants were obtained: nine for FGSG_01831, seven for FGSG_03599, and ten for FGSG_12160 (named as Δ01831, Δ03599, and Δ12160 respectively). The mutants were purified by single-spore isolation. The target gene replacement was confirmed by multiple PCRs.

### Colony Growth and DON Production Was Unaltered in Deletion Mutants Δ01831, Δ03599, and Δ12160

All of the mutants appeared to grow normally on V8 agar plates. To examine if toxin production was altered, the mutants were cultured in agmatine liquid media for 7 days, extracted, and DON concentrations were measured by GC/MS. However, none of the deletion mutants significantly affected DON production (data not shown).

### Deletion Mutants Δ01831, Δ03599, and Δ12160 Did Not Differ in FHB Spread

**Figure 3:**
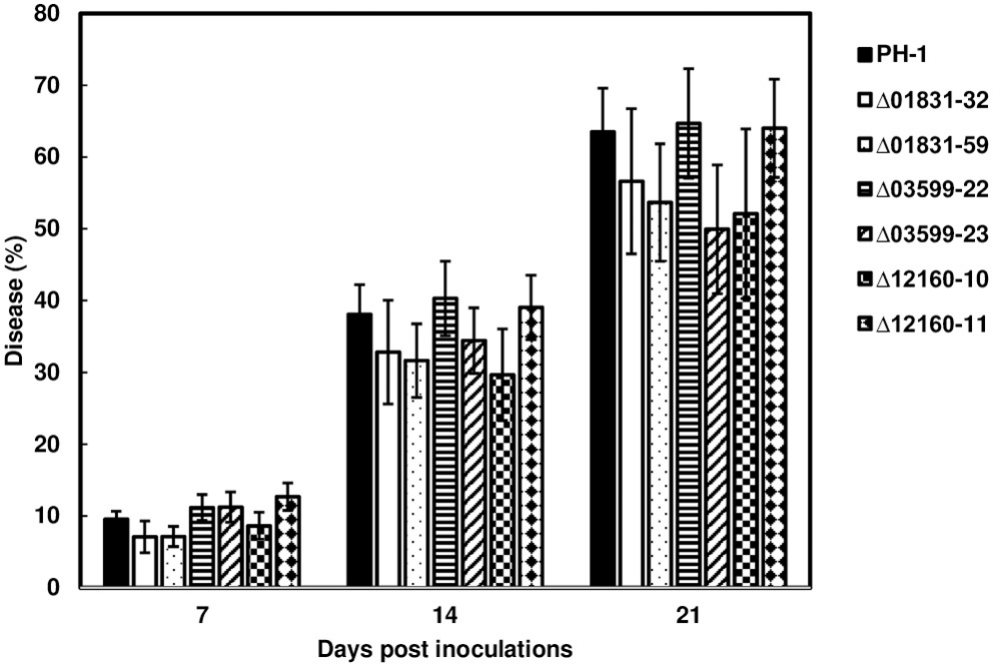
FHB spread from the effector mutants during wheat head infection. Point inoculations (10 μL spore suspension containing 1,000 conidia) were performed on wheat florets (cv. Norm) with *F. graminearum* wild-type PH-1 and mutants Δ01831-32 and 59, Δ03599-22 and 23, and Δ12160-10 and 11. FHB disease progression was measured by the percentage of florets infected at 7, 14, and 21 days post inoculation (dpi). Bars represent the average percentages and standard error of infected spikelets with each strain. Means at each timepoint were analyzed independently and compared to the control PH-1 by on one-way analysis of variance (ANOVA) and *Dunnett's test* using JMP (*N* = 20; *P* > 0.05).

To determine whether these effectors were associated with FHB spread in wheat heads, inoculum from the parent strain PH-1 and the mutant strains, Δ01831-32 and 59, Δ03599-22 and 23, and Δ12160-10 and 12, were point-inoculated in the susceptible wheat variety, Norm. FHB disease progression was evaluated over a 21-day period. FHB progressed similarly between PH-1 and mutants over the evaluation period. Statistical analysis showed that the effector mutants did not significantly affect FHB scores at the individual time points compared to control wheat heads inoculated with the parent strain PH-1 (Figure 3). Our data suggest that these effectors are non-essential for FHB spread.

### Δ01831 Mutants Reduce FHB Initial Infection

**Figure 4:**
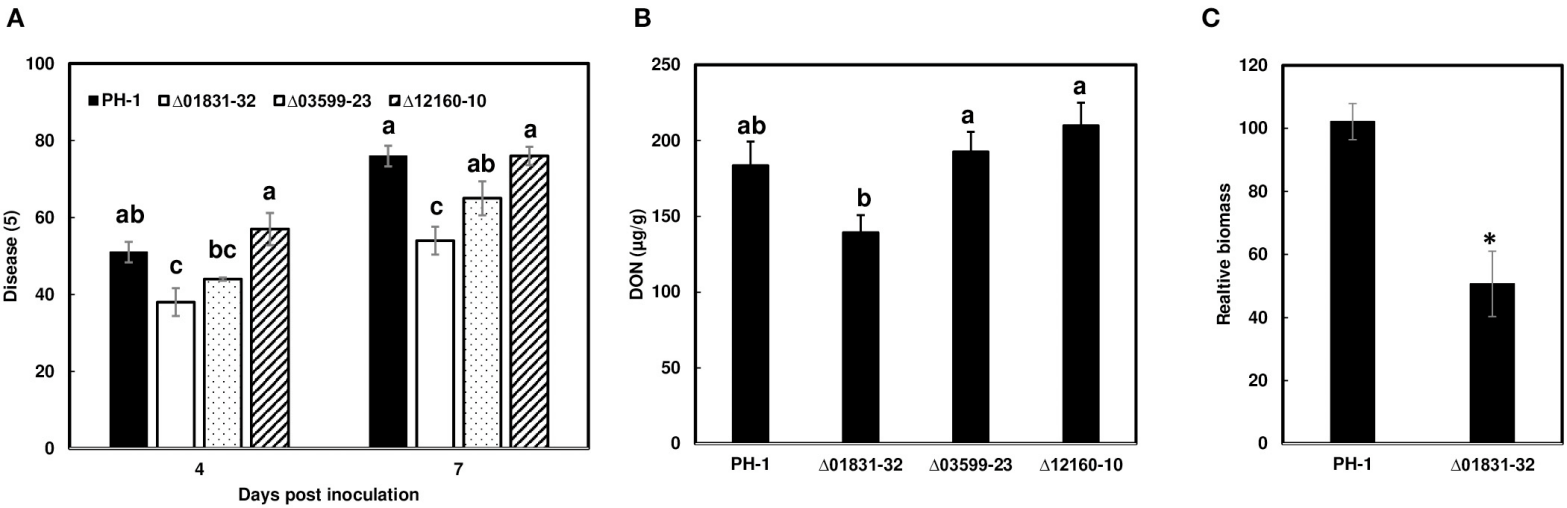
Impact of the effector mutants on initial infection, DON content, and fungal biomass on wheat heads. Inoculations were performed by immersing whole heads of wheat cv. Alsen in suspensions of *F. graminearum* PH-1 and mutants Δ01831-32, Δ03599-23, and Δ12160-10, respectively, (5 × 10^4^ conidia/mL). **(A)** FHB was scored as the percentage of florets with visual symptoms at 4- and 7-days post inoculation (dpi). Bars represent the average percentages and standard error of 24 inoculated heads with each strain. The statistical analyses were performed for each time point independently. **(B)** DON was extracted from tissues at 7 dpi and analyzed by GC/MS. **(C)** Comparison of fungal biomass relative to wheat tissue in infected wheat heads between PH-1 and mutant Δ01831-32. Relative biomass was calculated based on qPCR *Ct* values of fungal *TRI6* vs. the corresponding Ct values for the wheat gene GAPDH. Letters indicate significance based on one-way analysis of variance (ANOVA) and *Dunnett's test* using JMP. The asterisk (*) indicates significant difference at the *P* < 0.05 level by Student's *t*-test.

**Figure 5:**
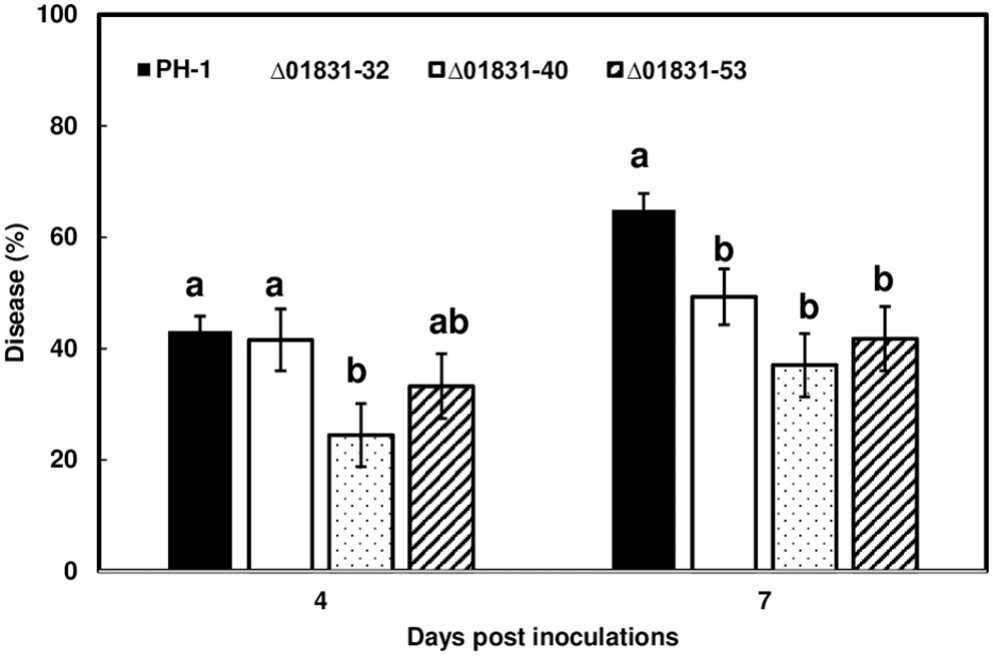
Reduction of initial infection caused by Δ01831mutants on wheat heads. Inoculations were performed by immersing whole heads of wheat cv. Alsen in suspensions of *F. graminearum* PH-1 and three Δ01831 mutants (32, 40, and 53), respectively (5 × 10^4^ conidia/mL). FHB was scored as the percentage of florets with visual symptoms at 4- and 7-days post inoculation (dpi). Bars represent the average percentages and standard error of 20 inoculated heads with each strain. The statistical analyses were performed for each time point. Letters indicate significance level at the *P* < 0.05 level by one-way analysis of variance (ANOVA) and *Dunnett's test* using JMP.

Due to challenges in distinguishing between initial infection and disease spread on the FHB susceptible variety Norm, we used the moderately resistant wheat variety Alsen to evaluate and compare the initial infection phenotypes of the mutant strains. The Δ01831-32 strain had 13% less infection at 4 dpi (*P* = 0.04), and 22% less infection at 7 dpi (*P* = 0.0001) in comparison to the control PH-1. Furthermore, there was 24% less DON (*P* = 0.057) and 50% less fungal biomass (*P* = 0.007) in wheat heads inoculated with the Δ01831 mutant compared to controls (Figure 4). In contrast, deletion mutants Δ03599 and Δ12160 did not significantly affect initial infection and DON production (Figure 4). To confirm the infection reduction resulted from deletion of FGSG_01831, three mutants (Δ01831-32, 40, and 53) were inoculated in replicated experiments. All three mutants significantly reduced infection at 7 dpi (Figure 5). These results indicate that FGSG_01831 contributes to *F. graminearum* initial infection and likely interferes with the type I resistance mechanisms in wheat.

### *F. graminearum* Effectors Suppress Chitin-Induced ROS Burst

**Figure 6:**
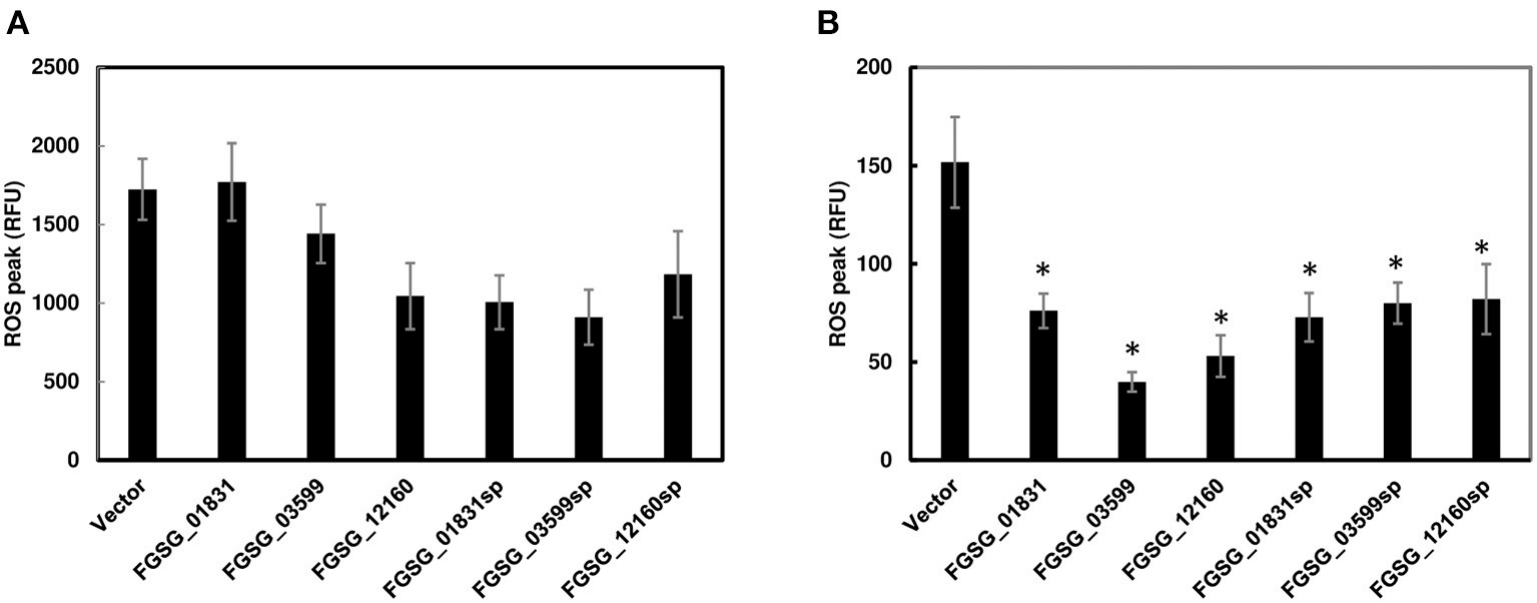
Reactive oxygen species (ROS) production in *N. benthamiana* leaves infiltrated with *Agrobacterium* strain GV2260 carrying *F. graminearum* effector genes or an empty vector. **(A)** ROS induced by flg22; **(B)** ROS induced by chitin. Luminescence was measured in 200 μL of assay solution (17 mM lumino, 1 μM horseradish peroxidase, and 100 nM flg22 or 200 μg/mL crab shell chitin) for 60 min. Leaves infiltrated with an empty vector served as positive controls. Bars represent the means of 12 replicates. Error bars represent the standard error of the mean (SEM). Statistical significance compared to *N. benthamiana* leaves infiltrated with an empty vector is indicated by asterisks (one-way ANOVA and Tukey *post-hoc* test, *P* < 0.05). The assays were repeated at least three times with similar results.

Since functional redundancy tends to mask the role of individual effectors in single-gene deletion mutants, we further investigated the potential function of these effector genes by transiently expressing individual effector genes with/without the signal peptide sequences in *N. benthamiana* via *Agrobacterium*-mediated transformation. However, none of the three effectors, including signal peptide FGSG_01831sp, FGSG_03599sp, and FGSG_12160sp or without signal peptide FGSG_01831, FGSG_03599, and FGSG_12160, elicited necrosis or programmed cell death when transiently expressed in *N. benthamiana* leaves. We further investigated if these effectors could suppress PTI. ROS production was measured in infiltrated leaf zones expressing each effector independently. Two days after infiltration, we removed leaf discs from infiltrated zones, and then treated them with bacterial flagellin peptide flg22 or crab chitin. None of them showed a significant reduction of ROS burst induced by flg22 (Figure 6A). In contrast, all three effectors with or without signal peptide significantly suppressed ROS production induced by chitin (Figure 6B). To ensure the ROS suppression was specific, an effector (FgNls1) targeting the plant nucleus was tested; however, no ROS suppression triggered by flg22 or chitin was observed (data not shown). These data indicate that these three effectors suppress chitin-induced ROS signaling mediated plant immunity.

### Suppression of Bax-Induced Cell Death by FGSG_01831 and FGSG_03599

**Figure 7:**
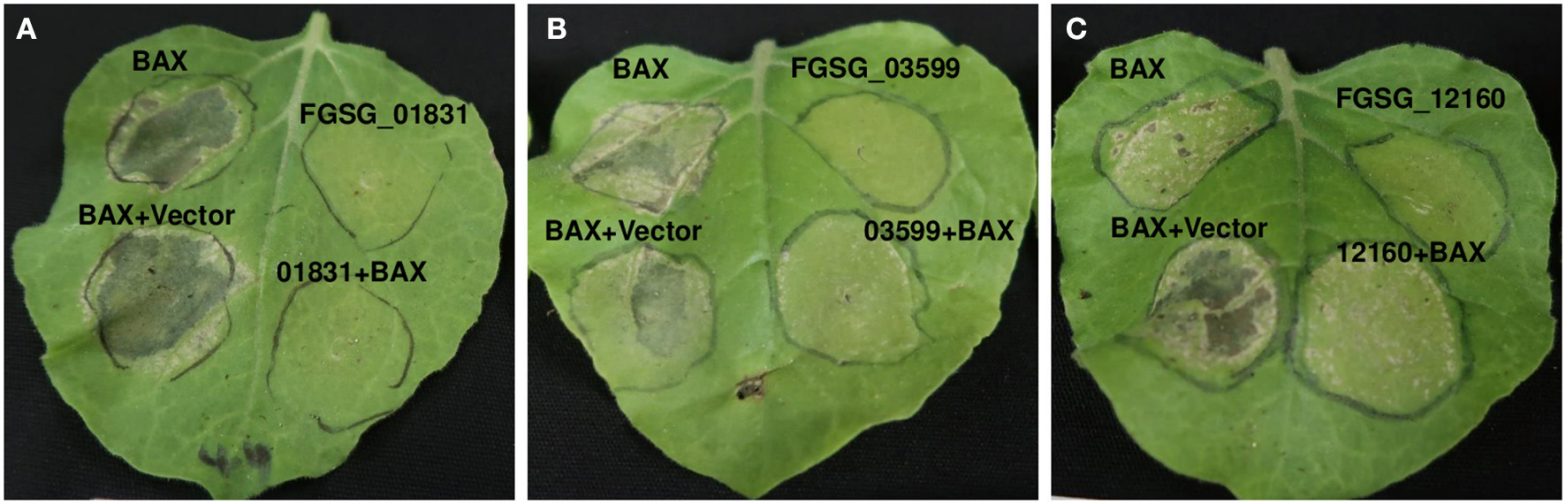
Suppression of Bax-induced cell death by *F. graminearum* effectors. **(A)** FGSG_01831, **(B)** FGSG_03599 and **(C)** FGSG_12160 *Agrobacterium* strain GV2260 carrying Bax was co-infiltrated in *N. benthamiana* leaves with each GV2260 strain carrying a *F. graminearum* effector or an empty vector. Each *A. tumefaciens* strain containing Bax, the respective effector gene, or an empty vector control was adjusted to OD_600_ = 1.0 then mixed in equal volumes. Cell death induced by Bax was visualized and imaged 5 days after infiltration. Six leaves from two plants for each effector were infiltrated. Three independent experiments were performed, and similar results were observed.

Since these effectors affected ROS production, we investigated whether they could also inhibit the cell death induced by Bax. Leaves expressing Bax showed cell death 3 days after infiltration, whereas none of the leaves infiltrated with any of the three effectors with signal peptide showed cell death (Figure 7). The leaves co-expressing Bax and FGSG_01831sp or FGSG_03599sp showed markedly reduced cell death, suggesting that both FGSG_01831sp and FGSG_03599sp were capable of suppressing Bax-induced cell death (Figures 7A,B). However, the leaves expressing FGSG_12160sp and Bax did not significantly suppress cell death induced by Bax (Figure 7C). In addition, similar phenotypes were observed when these effectors without signal peptide were expressed alone or co-expressed with Bax (data not shown). Taken together, our findings indicate that these three *F. graminearum* effectors may suppress plant immunity to promote FHB pathogenesis.

## Discussion

In the current study, we identified three *F. graminearum* effector genes that are highly induced during the first week of infection and revealed one of them, *FGSG_01831*, is involved in initial infection of wheat. Although *FGSG_01831* was not essential for disease establishment, deletion of this gene significantly reduced initial infection and DON contamination in wheat. Interestingly, while only deletion mutants of FGSG_01831 displayed differences in pathogenic fitness, all three effectors affected plant immunity and suppressed chitin-induced ROS burst via transient expression in *N. benthamiana*. Furthermore, when co-expressed with Bax, FGSG_01831 and FGSG_03599 markedly inhibited Bax-induced cell death. Although we demonstrated that each of these *F. graminearum* effectors affected plant immunity, the single-gene deletion mutants of FGSG_03599 or FGSG_12160 did not affect FHB. This may be due to gene function redundancy, compensation, or perhaps their minor role is masked by DON. The characterization of these effectors and their ability to affect plant immunity has improved our understanding of the complex of interactions that occur during FHB pathogenesis and underscore the difficulty in controlling FHB infection.

Pathogens secrete waves of effectors during plant infection and their expression was fine-tuned to the different infection stages (Gohari et al., 2015). In our study, a few effectors were induced in the very early stage (before 36 hpi). These effectors may aid in pathogen penetration and colonization. Expression of *TRI5*, the initial gene responsible for DON biosynthesis, initiated at 2 dpi and peaked at 3 dpi, and then gradually reduced (Figure 2). At least twelve *TRI* genes are involved in DON biosynthesis and regulation. The expression of many *TRI* genes, such as *TRI5, TR6, TRI101, Tri12*, etc., initiated at 48 h and peaked at 96 h after wheat head inoculation (Amarasinghe and Fernando, 2016). The expression of *FGSG_03599* displayed a similar expression pattern as *TRI5*. The expression of *FGSG_01831* and *FGSG_12160* were overlapped with *TRI* genes during initial infection. Interestingly, *FGSG_01831* displayed different induction patterns between the FHB susceptible Norm and the moderately resistant Alsen (Figure 2). Instead of showing an expression peak at 6 dpi in inoculated Norm, *FGSG_01831* expression remained highly induced from 3 to 7 dpi in Aslen. Although DON is less critical in initial infection compared to FHB spread, its accumulation may contribute to the shift from a biotrophic stage to a necrotrophic stage. Studies observed that Tri5 mutants grew 65–70% less than wild-type strain at 5 dpi and 7 dpi and suggested that DON facilitates *F. graminearum* colonization at the early stage of infection (Mentges et al., 2020). Therefore, investigation of the effector expression profiles in a *TRI5* deletion background may assist to elucidate the roles of effectors during FHB pathogenesis.

Many effectors have been identified in fungal pathogens, and their modes of action during pathogenesis have been characterized (Lo Presti et al., 2015). However, due to their functional redundancy, effector deletion mutants rarely result in significant disease reduction. For example, a study of 78 effector deletion mutants in *Magnaporthe oryzae* identified only one effector, M69, that contributed to virulence (Saitoh et al., 2012). In *F*. *graminearum*, disruption of xylanases, which were induced during plant infection, did not affect FHB level (Sella et al., 2013). Similarly, our study showed that many effector-encoding genes are induced during wheat head infection. However, deletion mutants of the three highly induced genes did not significantly affect FHB spread on wheat heads. It has been demonstrated that multiple effectors, such as AVR-Pik, AVR-Pia, and AVR1-CO39 in *M. oryzae*, target the same plant host protein to ensure successful infection (Bialas et al., 2018). This may be the case with *F. graminearum* and plant interactions, and further investigations are needed. In addition to functional redundancy of effectors, another obstacle is the disease evaluation, i.e., when counting florets with visual FHB symptoms, it is difficult to distinguish the minor or subtle changes of disease. To determine the minor changes caused by the mutants, more accurate detection methods are needed to compare the level of disease difference. On the other hand, Quarantin and associates proposed some genes as accessory virulence genes: disruption of an accessory virulence gene will not change the overall virulence of a fungal pathogen because the defective functions are compensated (Quarantin et al., 2019). The effectors that are highly induced during plant infection and are dispensable for FHB, such as *FGSG_03599* and *FGSG_12160*, could be considered as accessory virulence genes. It is worth noting that *FGSG_00060*, encoding a killer toxin, was not induced in our study. While a previous study showed that this gene was induced during FHB infection, deletion of FGSG_00060 did not reduce FHB but reduced seedling infection caused by *F. graminearum* (Lu and Edwards, 2018). Since *FGSG_03599* and *FGSG_12160* suppressed chitin-triggered ROS production in *N. benthamiana*, it is possible these effectors may play a role during infection of seedlings or other wheat tissues.

Sequence analysis suggests that *FGSG_01831* encodes a Class II hydrophobin. It shares high homology with Mhp1 from the rice blast fungus *M. grisea*, and the mutants of Mhp1 reduce appressorium formation and pathogenicity (Kim et al., 2005). The *F. graminearum* genome contains four Class I hydrophobins, and two of them (FGSG_01764 and 09066) have been demonstrated to display characteristics of hydrophobins, such as attachment to hydrophobic surface and penetration through the water-air interface (Quarantin et al., 2019). However, mutants of FGSG_01831 did not affect water-repellence (Quarantin et al., 2019). Similarly, we found that Δ01831 mutants displayed a similar water- and detergent- repellence as the wild-type strain (data not shown). Therefore, experimental data do not support the hypothesis that FGSG_01831 functions as a typical hydrophobin.

Although Δ01831 mutants did not significantly affect FHB spread in Norm, the mutants significantly reduced initial infection in moderately resistant wheat Alsen. We showed that the expression of *FGSG_01831* initiated at 2 dpi, gradually increased, and peaked (272-fold) at 6 dpi in Norm. Whereas in Alsen, FGSG_01831 transcripts remained abundant from 3 dpi (175-fold) to 7 dpi (243-fold). This suggests that FGSG_01831 is important during the first week of infection. In addition, significantly less fungal biomass and DON were detected in Alsen wheat heads inoculated with Δ01831 compared to wild-type PH-1. On the contrary, Quarantin and associates found that FGSG_01831 mutants did not affect FHB initial infection and spread on wheat, but neither fungal biomass nor DON was examined (Quarantin et al., 2019). The different results may be due to the wheat varieties used in the experiments. Quarantin and associates performed dip inoculation on a susceptible wheat variety in which disease rapidly spread making it difficult to observe differences in initial infection (Quarantin et al., 2019). We conducted dip-inoculation on the moderately resistant variety Alsen, which contains QTL (*Fhb1* and *Fhb5*) that confer resistance to initial infection and spread of FHB (Frohberg et al., 2006; Bokore et al., 2017). When disease progression is constrained by host resistance, the subtle roles of effectors in initial infection may be more apparent. Typically, hydrophobins from filamentous fungi coat the surface of conidia, spores, hyphae, and fruiting bodies to confer water-repellence and protect fungi from the aqueous environment. Hydrophobins have been shown to play an important role during fungal attachment and colonization of the host, but only a few studies have demonstrated that a hydrophobin can directly interfere with plant immunity (Huang et al., 2015; Guzmán-Guzmán et al., 2017). *N. benthamiana* leaves expressing FGSG_01831 showed reduced ROS production induced by chitin. These observations suggest that FGSG_01831 is involved in suppression of chitin-induced plant immunity during *F. graminearum* infection. When FGSG_01831 and Bax were co-expressed, FGSG_01831 suppressed cell death induced by Bax. Nevertheless, it is unclear why only the FGSG_01831 mutants resulted in the observed phenotype of compromised initial infection while the other effectors with similar function in inhibiting ROS and suppressing cell death are not. Further investigations are needed to determine the precise mode of action of FGSG_01831 during *F. graminearum* pathogenesis.

FGSG_03599 contains a CFEM domain that mediates signal transduction and surface adhesion during host-pathogen interactions (Kulkarni et al., 2003). For example, a CFEM protein, Pth11 from *Magnaporthe grisea*, is involved in appressoria formation and host colonization (Dezwaan et al., 1999). In the *F. graminearum* genome, six CFEM-containing proteins were identified including FGSG_00588, 02077, 02181, 03573, 03599, and 08554 (Lu and Edwards, 2016). Among them, none of transcripts of *FGSG_02181* or *03573* were detectable *in vitro* or *in planta* infection, whereas *FGSG_ 00588, 02077*, and *08544* were constitutively expressed *in vitro* or *in planta* but not significantly induced during wheat infection in comparison to expression in axenic culture. Only the expression of *FGSG_03599* was induced during wheat head infection (Lu and Edwards, 2016). In agreement with the previous study, we found that the expression of *FGSG_03599* peaked in Norm (200-fold) and Alsen (110-fold) at 3 dpi after the *F. graminearum* infection. However, disruption of FGSG_03599 did not significantly reduced FHB spread or initial infection. Since the chitin induced ROS and Bax induced cell death were similarly inhibited in *N. benthamiana* leaves expressing *FGSG_03599* and *01831*, our results suggest that FGSG_03599 plays a subtle role during *F. graminearum*-plant interactions. It's also possible that the role of FGSG_03599 was masked by DON because it displayed a similar expression pattern with *TRI5*. The potential role of FGSG_03599 in FHB pathogenesis needs further investigation by generation of double deletion mutants of FGSG_03599 and *TRI5*. In addition, mutants of *FGSG_02077*, which was induced during coleoptile infection, caused reduced lesion sizes on wheat coleoptiles (Zhang et al., 2012). It is of interest to determine whether FGSG_03599 plays a role during coleoptile infection and whether double-gene deletion mutants of FGSG_02077 and 03599 affect FHB pathogenesis.

FGSG_12160 has the characteristics of a glycosyl hydrolase 61 (GH61). A Cel61B protein belonging to GH61 was identified from *Hypocrea jecorina*, and it showed a potential endoglucanase function (Karkehabadi et al., 2008). The expression of *FGSG_12160* was induced over 4,000-fold in Norm and 643-fold in Alsen at 3 dpi (Figure 2). Surprisingly, the deletion mutants of this gene did not significantly affect FHB development by point or dip inoculations. A prior study showed that one GH61 member, Pelg1 from the tomato pathogen *Pyrenochaeta lycopersici*, was induced during host infection, but no mutants were generated to determine its involvement in pathogenesis (Valente et al., 2011). Although no homologs of FGSG_12160 were identified at the protein level by searching the *F. graminearum* PH-1 genome, the GH61 family members may share similar structure and enzyme function without high level protein similarity. A group of GH61 enzymes (FGSG_03632, 03695, 08011, and 04773) showed high expression at 16 h during the wheat coleoptile infection (Zhang et al., 2012). However, it is unknown whether these GH61 members are induced during FHB development. Therefore, it will be interesting to determine their expression during wheat head infection, and their effect on FHB by a single gene knockout and by simultaneously knocking out some of them.

We demonstrated that all three effectors (FGSG_01831, FGSG_03599, and FGSG_12160) significantly reduced ROS burst induced by chitin but had no significant reduction on ROS induced by flg22. Flg22 peptide is derived from bacterial flagellin, whereas chitin oligosaccharides are components of fungal cell walls. These two PAMPs are structurally different and perceived by different receptor complexes. Our results suggest that these three effectors are only involved in the chitin-mediated signaling system. In contrast, we previously demonstrated that *F. graminearum FgARB93B* suppresses ROS induced by both flg22 and chitin (Hao et al., 2019). Similarly, previous reports have shown that some effectors from *Pseudomonas syringae* differently suppressed ROS induced by flg22 in comparison to chitin, whereas others suppress ROS induced by flg22 and chitin in a similar manner (Gimenez-Ibanez et al., 2018). However, it is unknown how the fungal and bacterial effectors evolve the specificity to suppress ROS triggered by different elicitors.

Many bacterial, oomycete, and fungal effectors elicit cell death in *N. benthamiana*. Cell death has been observed for multiple effectors from *Zymoseptoria tritici* when transiently expressed in *N. benthamiana* with a signal peptide, whereas no cell death or weaker response was detected without a signal peptide (Kettles et al., 2017). However, none of the three effectors, with or without a signal peptide, from *F. graminearum* induced cell death or leaf chlorosis when transiently expressed in *N. benthamiana*. So far, only a few *F. graminearum* secreted proteins have been reported to induce necrosis. Of them, mutants of xylanases did not affect FHB development (Sella et al., 2013). Cell death is associated with effector recognition by R proteins. Since tobacco is not a host for *Fusarium*, it is possible that no corresponding R gene interacts with *F. graminearum* effectors to induce cell death. In contrast, we found that expression of FGSG_01831 and FGSG_03599 almost completely suppressed Bax-induced cell death. Many studies have shown the suppression of Bax-induced cell death by effectors from bacterial, fungal and oomycete pathogens. Our prior study identified that *F. graminearum FgARB93B* suppresses ROS associated plant immunity and Bax-induced cell death (Hao et al., 2019). These results indicate that *F. graminearum* employs multiple effectors to suppress PTI and promote infection. Further investigations are needed to understand the mode of action of these effectors and their synergistic role with one another or DON to promote FHB.

In summary, we identified three highly induced *F. graminearum* effector-coding genes that affect plant immunity during wheat head infection. However, only FGSG_01831 was shown to independently contribute to initial infection of wheat in a manner that resulted in a compromised disease phenotype when this gene was disrupted. Therefore, our results suggest that these three effectors are involved in FHB pathogenesis. The interactions between *F. graminearum* and its host plants are complicated, and due to the large number of effectors including DON, it is challenging to functionally characterize each of them individually. It is likely that multiple gene deletions are needed to dissect the roles of effectors during FHB infection.

## Data Availability Statement

The original contributions presented in the study are included in the article/Supplementary Materials, further inquiries can be directed to the corresponding author/s.

## Author Contributions

GH: conceived and designed the experiments and wrote the manuscript. TU, HT, GH, SM, and MV: performed the experiment. GH and HT: analyzed the data. All authors edited, reviewed, and approved the manuscript.

## Conflict of Interest

The authors declare that the research was conducted in the absence of any commercial or financial relationships that could be construed as a potential conflict of interest.
