# Supplementary material for: Characterization of Three Fusarium graminearum Effectors and Their Roles During Fusarium Head Blight
Source: Front Plant Sci. 2020 Nov 30;11:579553. doi: 10.3389/fpls.2020.579553 (PMC7734257; doi:10.3389/fpls.2020.579553)
Supplement: Supplementary file 1 [file Table_1.docx]

**Supplementary Material**

**Supplementary Table S1. RT-PCR primers used in this study**

|  | |  |  |
| --- | --- | --- | --- |
| **Gene name** | | **Primer sequence** |  |
|  | |  |  |
| FGSG_00060-F | | GGATCTATCTGCGCCTTCTTC |  |
| FGSG_00060-R | | TGCCTTCTTCAGTGGGAATAC |  |
|  | |  |  |
| FGSG_00569-F | | GTGCGGCAAAGCGTTTAAT |  |
| FGSG_00569-R | | CTTTGGCTTTGGTGGTGTTC |  |
|  | |  |  |
| FGSG_01831-F | | CTTGGAGCCGTTGTCTCTG |  |
| FGSG_01831-R | | CAGTAGCACAGCACTGAGAAG |  |
|  | |  |  |
| FGSG_02063-F | | ACAAGACCTCGACTTCACAAC |  |
| FGSG_02063-R | | CAGGAACGAAACCGGAAGTAG |  |
|  | |  |  |
| FGSG_03581-F | | GACTTTACCAACTGGGACGAT |  |
| FGSG_03581-R | | TGCTGTTCTGCTCAAGGTAG |  |
|  | |  |  |
| FGSG_03599-F | | CCGAGACGTCAGCCATTC |  |
| FGSG_03599-R | | TCCACAGTTACCGACAACAC |  |
|  | |  |  |
| FGSG_04074-F | | CGATGACCGTGACAACTTCA |  |
| FGSG_04074-R | | CAGAGACACAGTCCTTGTTACC |  |
|  | |  |  |
| FGSG_04239-F | | TACTGGCTTTGGAGGGTACT |  |
| FGSG_04239-R | | CATCCGATCTCCCTGATTCTTC |  |
|  | |  |  |
| FGSG_04661-F | | TTCAATGGGCCCAAGACTAC |  |
| FGSG_04661-R | | GGCACTGGTGATAACTTTCTTTC |  |
|  | |  |  |
| FGSG_04805-F | | CTCGCCAATCCCATCGATAC |  |
| FGSG_04805-R | | AGTGCTGTCCCAGAACTTAAC |  |
|  | |  |  |
| FGSG_05341-F | | GTTGCTACGCCCACTAAAGA |  |
| FGSG_05341-R | | GCAGACCTTGACCGATTCA |  |
|  | |  |  |
| FGSG_05714-F | | CCACACACAGCTTCACTACTT |  |
| FGSG_05714-R | | CTGGGACGCCAGTCTTTAC |  |
|  | |  |  |
| FGSG_06712-F | | CAAGCGACAGGAAGACAAGA |  |
| FGSG_06712-R | | ATGTGTTATCAGCACCCTCAG |  |
|  | |  |  |
| FGSG_06993-F | | TGCTGCTTCTTGGACTACTG |  |
| FGSG_06993-R | | CTCTTGGACTGGTTGACGTT |  |
|  | |  |  |
| FGSG_08210-F | | CTACCACAAGACCGACAAGAC |  |
| FGSG_08210-R | | TAGATCCCTGAGCACCAGTAA |  |
|  | |  |  |
| FGSG_09570-F | | CCTGGCTCTTGCTGTTCTATC |  |
| FGSG_09570-R | | GGGTTCAATGGGCTTGTAGT |  |
|  | |  |  |
| FGSG_10206-F | | CACTGCTGCTTCACTCTACAT |  |
| FGSG_10206-R | | GCGAGTTGGACAGGTCAAT |  |
|  | |  |  |
| FGSG_11205-F | | CTCTTGGGTTGAGTTCGCTAC |  |
| FGSG_11205-R | | CCATCCACCGCCAATGATAA |  |
|  | |  |  |
| FGSG_11318-F | | ACCACCAGCTTGTCCAATAC |  |
| FGSG_11318-R | | GTTCCACTCGAATCTCCTGATG |  |
|  | |  |  |
| FGSG_12160-F | | TGGTTGCGATGCTTGGAT |  |
| FGSG_12160-R | | GATAACGGAAGAAGCCCAGTT |  |
|  | |  |  |
| FGSG_12622-F | | CACAGAATGGCCACCTATTGA |  |
| FGSG_12622-R | | CCCAAGACTATCGGGACAAAC |  |
|  | |  |  |
| FGSG_13782-F | | GCCCTCCCTATAACTTTCGATG |  |
| FGSG_13782-R | | AAGAGCACCTTGTCTTGTCC |  |
|  | |  |  |
| FGSG_13952-F | | TATGGGTGTCGAGGTTGTTG |  |
| FGSG_13952-R | | CCTTGGCCGACACATTCT |  |
|  | |  |  |
| FGSG_09530-β-tubulin-F | | TCCAGGGTTTCCAAATCACC |  |
| FGSG_09530-β-tubulin-R | | GGAACGACGGAGAAAGTTGC |  |
|  | |  |  |
|  | |  |  |
| **Supplementary Table S2.** **Primers used for transient expression and mutagenesis** | | | |
|  |  | | |
| **Gene name** | **Primer sequence** | | |
|  |  | | |
| **Transient expression** |  | | |
|  |  | | |
| FGSG_01831-attb1 | GGGGACAAGTTTGTACAAAAAAGCAGGCTggATGAAGTTCTCACTCGCCGC | | |
| FGSG_01831MP-attb1 | GGGGACAAGTTTGTACAAAAAAGCAGGCTggATGGCCCTTCCCGCCAACGAG | | |
| FGSG_01831-attb2 | GGGGACCACTTTGTACAAGAAAGCTGGGTgGTAGTCCTGGACACCAGTAG | | |
|  |  | | |
| FGSG_03599-attb1 | GGGGACAAGTTTGTACAAAAAAGCAGGCTccATGCAGTTCACCACCTCCTTC | | |
| FGSG_03599MP-attb1 | GGGGACAAGTTTGTACAAAAAAGCAGGCTccATGCCACAGGCCGCACCAG | | |
| FGSG_03599-attb2 | GGGGACCACTTTGTACAAGAAAGCTGGGTgGTAAGCACAGGCAGTGCAGA | | |
|  |  | | |
| FGSG_12160-attb1 | GGGGACAAGTTTGTACAAAAAAGCAGGCTggATGCATATCTCTCTTTCTCTGCTTT | | |
| FGSG_12160MP-attb1 | GGGGACAAGTTTGTACAAAAAAGCAGGCTggATGCACGGCTACGTCGAGAC | | |
| FGSG_12160-attb2 | GGGGACCACTTTGTACAAGAAAGCTGGGTgCTAGCACTTGAACACAGCAGG | | |
|  |  | | |
| **Mutagenesis** |  | | |
|  |  | | |
| OSC-F | CTAGAGGCGCGCCGATATCCT | | |
| OSC-R | CGCCAATATATCCTGTCAAACACT | | |
| Hyg-R210 | GACTTCCATACCACAGGATGTTATC | | |
| Hyg-F850 | AGAGCTTGGTTGACGGCAATTTCG | | |
|  |  | | |
| FGSG_01831-attb2r | GGGGACAGCTTTCTTGTACAAAGTGGAAATGAGGTGGCTGGATGGAAAT | | |
| FGSG_01831-attb1r | GGGGACTGCTTTTTTGTACAAACTTGTGATGTGCTTTGAGGATGTGAATG | | |
| FGSG_01831-attb4 | GGGGACAACTTTGTATAGAAAAGTTGTTGTGTCACGAGTTTGTGACCTTGTAT | | |
| FGSG_01831-attb3 | GGGGACAACTTTGTATAATAAAGTTGCCAAGATGCCTTCCAGTTCTAC | | |
| FGSG_01831-ORF-5' | CTTGGAGCCGTTGTCTCTGCC | | |
| FGSG_01831-ORF-3' | TAGTCCTGGACACCAGTAGG | | |
| FGSG_01831-up | AGCATGCCACTGATGACGATG | | |
| FGSG_01831-down | CCTGCTTCACCGTCAACCGTG | | |
|  |  | | |
| FGSG_03599-attb2r | GGGGACAGCTTTCTTGTACAAAGTGGAACTCCACCAATGCCCATAACA | | |
| FGSG_03599-attb1r | GGGGACTGCTTTTTTGTACAAACTTGTCAATCCAACACACAAACATCCA | | |
| FGSG_03599-attb4 | GGGGACAACTTTGTATAGAAAAGTTGTTTTGATTACAGGCTCGTGGGA | | |
| FGSG_03599-attb3 | GGGGACAACTTTGTATAATAAAGTTGTTGGACAGTAGGGAGAGATAGG | | |
| FGSG_03599-ORF-5' | TCACCACCTCCTTCATCGTCG | | |
| FGSG_03599-ORF-3' | CTAAGCACAGGCAGTGCAGACG | | |
| FGSG_03599-up | GCCTGTATCTCCGAACGATCT | | |
| FGSG_03599-down | GAACGATCGAACTGCCAGTAG | | |
|  |  | | |
| FGSG_12160-attb2r | GGGGACAGCTTTCTTGTACAAAGTGGAAAGGTTGTGATGCTGAAGAGTAG | | |
| FGSG_12160-attb1r | GGGGACTGCTTTTTTGTACAAACTTGTGATGGGTATGTTTGAGATGGTATTG | | |
| FGSG_12160-attb4 | GGGGACAACTTTGTATAGAAAAGTTGTTCTCAGCATTGATTCGCTTGTAG | | |
| FGSG_12160-attb3 | GGGGACAACTTTGTATAATAAAGTTGCTAGACCTTGGACGTGTCATTC | | |
| FGSG_12160-ORF-5' | ATGCATATCTCTCTTTCTCTGCTTTG | | |
| FGSG_12160-ORF-3' | CTAGCACTTGAACACAGCAGG | | |
| FGSG_12160-up | ACTTAAAGAGCCTGCACCTAAC | | |
| FGSG_12160-down | CCCAAGGGTTCGGAAAGTTTAATA | | |
|  |  | | |
| For copy number |  | | |
|  |  | | |
| Hyg-RT-F | CCGCAAGGAATCGGTCAATA | | |
| Hyg-RT-R | GGTGTCGTCCATCACAGTTT | | |
| FgTRI6-RT-F | TAACCACATCGTCGGGACTG | | |
| FgTRI6-RT-R | GCCGACTTCTTGCAGGTCTT | | |
|  |  | | |
|  |  | | |
|  | | |  |

**Supplementary Figure 1** Gene expression profiles of selected effectors down regulated during wheat head infection. Wheat heads were collected at 3, 6, 12, 24, 36 h and 7 d after whole head dip inoculation. Three biological replicates, each replicate containing two heads, were collected at each time point and used for RNA isolation and cDNA synthesis. Fungal β-tubulin was used as an internal control for transcript normalization. Fold changes of gene expression were relative to axenic culture, which was grown on V8 plates for seven days. Relative expression profiling *in planta* was performed with three biological replicates.
